# Pre-referral stabilization and compliance with WHO guidelines for trauma care among adult patients referred to an urban emergency department of a tertiary referral hospital in Tanzania

**DOI:** 10.1186/s12873-019-0237-2

**Published:** 2019-02-28

**Authors:** Nanyori J. Lucumay, Hendry R. Sawe, Amour Mohamed, Erasto Sylvanus, Upendo George, Juma A. Mfinanga, Ellen J. Weber

**Affiliations:** 1 Emergency Medicine Department, Muhimbili University of Health and Allied Sciences, P.O. Box 65001, Dar es Salaam, Tanzania; 2 Emergency Medicine Department, Muhimbili National Hospital, Dar es Salaam, Tanzania

**Keywords:** Initial stabilization, Trauma patients, Pre-referral, Emergency care

## Abstract

**Background:**

The outcomes of trauma are considered to be time dependent. Efficient and timely pre-referral stabilization of trauma patients has been shown to impact survival. Tanzania has no formal pre-hospital or trauma system. World Health Organisation has provided a set of standards for initial stabilization of trauma patients according to the level of the hospitals. We aimed to describe pre-referral stabilization provided to adult trauma patient referred to the national referral hospital and compliance with World Health Organisation guidelines.

**Methods:**

This prospective observational cross-sectional study was conducted at the Emergency Medicine Department of Muhimbili National Hospital (EMD-MNH), between July 2017 and December 2017. Eligible patients were adults with head injury and extremity injury ≥18 years who were referred from a peripheral hospital and had a referral note. Research assistant enrolled patients using structured case report form clinical information, and initial stabilization received at the referring hospital. Primary outcome was the proportion of patients who had initial stabilization performed according to World Health Organisation recommendation.

**Results:**

We enrolled 368 (29% of eligible patients), the median age was 34 years (Interquartile range 26–44 years), and 281 (76%) were male. Overall 69% of referred patients arrived at the EMD more than 24 h after injury. Of those enrolled, 50 (13.6%) patients had received at least one stabilization intervention prior to transfer to MNH. Among 206 patients with extremity injuries, splinting was inadequate or missing in all cases; No patients with head injury received cervical spine protection. Among patients referred from a health center, 26.9% received an initial stabilization, while stabilization procedures were administered to 13.2% of those from district hospitals, and 10% of those from regional hospitals.

**Conclusions:**

In this urban public emergency department in Tanzania, majority of trauma patients were referred from lower health facilities after 24-h of injury. Most did not receive initial trauma stabilization as recommended by the World Health Organisation guidelines. Future studies should identify barriers to pre-referral stabilization of adult trauma patients.

## Background

Trauma is a leading cause of death and disability globally, accounting for more deaths than human immunodeficiency virus (HIV), malaria and tuberculosis combined [1–3]. Injury contributes about 5.8 million deaths worldwide, and it is estimated that 90% of trauma- associated mortality occurs in Low and Middle Income Countries (LMIC) [4, 5], increasing the overall burden of disease in these countries [3, 4, 6].

The incidence of trauma in LMIC has increased substantially [8–10]. In Tanzania, deaths from road traffic accidents accounts for over 40 % of all injuries [11]. For every death occurring due to road traffic accident there are fifty more survivors with permanent disability and deformity [4]. Despite this burden of trauma in Tanzania, the trauma system and preparedness for caring for trauma cases is inadequate, further contributing to death and disability [12].

In Tanzania, there are different levels of hospitals ranging from the health centers with limited resources and staffed by non-physicians with a diploma in clinical medicine, to the regional hospitals with more resources and specialist care. There is no formal pre-hospital system, thus trauma patients are brought to a nearby hospital by bystanders, relatives, or police who don’t have the knowledge to handle these patients appropriately. Moreover, local hospitals are not necessarily capable of caring for these trauma patients. As in other LIMC, emergency medicine is a new field (and few hospitals have emergency departments (ED), and only “casualty” units, who largely triage patients. Across the country there is only one full capacity trauma care centre the Muhimbili Orthopaedic Institute that is located in Dar es salaam and cares for all trauma patients with orthopaedic, trauma and neurosurgical cases. The Muhimbili National Hospital Emergency Department (MNH ED) receives these trauma patients in transfer from the district and regional hospitals in the city and outside the city.

Initial stabilization in trauma patients is defined by the World Health Organisation (WHO) essential trauma care as services provided to any trauma patient to prevent death or disability [2]. This includes recognition and correction of airway obstruction, breathing compromise and circulatory compromise at all hospitals. Depending on the level of hospital, additional stabilization measures are recommended [13]. The WHO essential trauma care guideline categorizes human and physical resources (infrastructure, equipment and supplies) that are necessary for provision of care at different levels of health facilities. The lower level health facilities are expected have less health care providers, and hence perform fewer interventions as compared to the higher-level health facilities.

It is unclear how well referring hospitals in Tanzania are able to comply with the WHO recommendations for initial stabilization for trauma patients. In order to improve the care of these trauma patients we conducted a study to determine the types of initial stabilization/intervention done for these patients and evaluate it in terms of WHO recommendations.

## Methods

### Study setting

This prospective observational study was conducted at Muhimbili National Hospital (MNH) a tertiary referral hospital located in Dar es salaam, Tanzania. MNH is the main national referral hospital that has a bed capacity of 1500. The Emergency Department (ED) is the main receiving department for all undifferentiated patients presenting with acute illnesses and injuries. The emergency department (ED) receives approximately 50,000 patients per year, and 80% are admitted. Trauma accounts for approximately 25% of all patients. Within MNH compound, there are two independent but connected institutions: the Jakaya Kikwete Cardiac Institute (designed for management of medical and surgical cardiac conditions) and Muhimbili Orthopaedic Institute (dedicated to care of orthopaedic and traumatology). Injured patients who require orthopaedic or neurosurgical admissions are transferred to the Muhimbili Orthopaedic Institute.

### Participants

All adult trauma patients’ ≥18 years with head injury, and/or extremity injuries (including pelvic fractures) received at MNH-ED with a referral note from a peripheral hospital were eligible to participate in the study. Patients who also had visceral injuries were excluded.

### Study protocol

A research assistant consecutively enrolled patients on a 12-h period on randomly selected days and alternate nights to ensure representativeness of the EMD trauma population. For consenting patients, the research assistance used a case report form (CRF) to document the required information including demographics, referring hospital, mode of arrival, mechanism of injury, vital signs, documented vital signs, physical examination, diagnosis, initial management/initial stabilization received at the referring hospital. To determine initial stabilization performed at the referring hospital, the research assistant reviewed the referral note, and asked the treating physician if, based on the appearance and condition of the patient on arrival, any other initial stabilization had been performed.

### Outcomes

The primary outcome of interest was the number and type of appropriate initial stabilization measures performed for these patients according to WHO recommendation for the particular injury. The secondary outcome was to determine the proportion of stabilization per level of referring hospital. There were no prior studies evaluating the frequency with which stabilization measures are performed at referring hospitals. Sample size was therefore estimated based on the proportion of completeness of documentation (30%) in a study by Mulunesh conducted in Ethiopia [14]. This gave a minimal sample size of 325.

### Data analysis

Descriptive data was entered into a online data Capture Software (REDCap version 7.2.2, Vanderbilt, Nashville, TN, USA) and the data was exported to IBM SPSS for analysis. Quantitative data was summarized using descriptive statistics.

## Results

### Patients demographics

During the study period, 2947 trauma patients presented to the ED. Of these, 1251 were adults transferred from an outlying hospital with referral notes and 368 (29%) of patients had head injury, extremity injury or both. After exclusion, there were 114 patients with isolated head injury, 206 with extremity injury and 48 with polytrauma. The median age was 34 years (Interquartile rage 26–44 years), and 281 (76%) were male. The majority of patients (69%) arrived more than 24 h after the injury. Patients were referred from six different levels of hospital; health centers, district hospitals, regional hospitals, private hospitals military and police hospitals, with the majority 261 (70%) referred from district hospitals (Table 1).

**Table 1:** Patients characteristics

	Number *N* = 367	Percentage %
Sex^a^		
Male	281	76.4
Female	86	23.4
Age Group	N	%
18–30 Years	155	42.1
31–45 years	132	35.9
46–60 years	42	11.4
> 60 years	39	10.6
Arrival to EMD	N	%
< 1 h to 24 h	114	31
> 24 h	254	69
GCS on arrival	N	%
GCS 3–8	14	3.8
GCS 9–12	13	3.5
GCS 13–14	31	8.4
GCS 15	289	78.5
Referring facility	N	%
Public Health centre	28	7.6
District hospital	261	70.9
Regional hospital	52	14.1
Private hospital	15	4.1
Military and police hospitals	12	3.2
Disposition	N	%
Discharged home	15	4.1
Admitted	18	4.9
Transferred to trauma center^b^	335	91

^a^ *One patient missed gender classification* ^b^ *Muhimbili Orthopaedic Institute*

### Mechanism of injury

Motorcycle accidents were the most common mechanism of injury, and far more common than other types of motor vehicle accidents (Table 2). Falls and pedestrians hit by motor vehicles were the second and third most common mechanisms. .

**Table 2:** Mechanism of injury

Mechanism of injury	N (%)	%
Motorcycle	104	28.3
Fall	85	23.1
Pedestrian	65	17.7
Other	49	13.3
Unknown	42	11.4
MVA	23	6.3

### Distribution of injury mechanism by age group

Motorcycle injuries accounted for half of the injuries 52(50%) in 18-30 years age group while falls were the primary mechanism in those over 60 (Table 3).

**Table 3:** Mechanism of injury by age group

Age in years	OVERALL	18–30	31–45	46–60	> 60
N	368 (100%)	155	132	42	39
Motorcycle n (%)	104 (28.3)	52 (50)	34 (32.7)	15 (14)	3 (2.9)
Fall n (%)	85 (23.1)	22 (25.9)	25 (29)	12 (14)	26 (30.6)
Pedestrian n (%)	64 (17.4)	24 (37.5)	27 (42)	8 (12.5)	6 (9.4)
Other n (%)	49 (13.3)	24 (49)	24 (49)	1 (2)	0
Unknown n (%)	42 (11.4)	21 (50)	15 (36)	3 (7)	3 (7)
MVA n (%)	23 (6.3)	12 (52)	7 (30)	3 (13)	1 (4)

### Pre-referral stabilization

Overall, 50 (13.6%) patients received at least one stabilization procedure at the outlying hospital. Of the 114 patients with head injury, 16(14%) received one or more stabilization interventions recommended by the WHO essential trauma care guideline. Of the 206 with extremity injuries, 14(6.8%) received at least one recommended stabilization intervention. (Table 4) Notably, no patients with head injury had cervical spine stabilization, and patients with extremity injuries had no or inadequate splints. Among those with extremity injuries, 6 patients (3.4%) had documented admnistration of pain medicine. Of 14 pts. with airway compromise, none arrived with or had a documented oral airway or intubation, however three of them had suctioning performed. The proportion of patients with interventions varied by hospital level. Of patients referred from a health center level (least resourced) to MNH ED, 27% had received at least one stabilization intervention. Among those arriving from the district hospitals, 13% had interventions, and those from regional hospitals, 31.3%. 22% of patients transferred from a military or police hospital had interventions.

**Table 4:** Initial stabilization per referring level

Characteristic	HC^a^	DH^a^	RH^a^	PRVT^a^	Other^a^
Head injury (*N* = 114)	(*N*= 9)	(*N*= 78)	(*N*= 22)	(*N*= 4)	(*N*= 1)
Mannitol	0	0	0	0	0
Iv fluid	1	9	0	0	0
Dextrose	1	0	0	0	0
Pain med	0	5	0	0	0
Burr hole	0	0	0	0	0
Neck color	0	0	0	0	0
Extremity injury (*N* = 206)	(*N*= 12)	(*N*= 150)	(*N*= 28)	*N*= (11)	(*N*= 5)
Splinting	0	0	0	0	0
Iv fluid	1	6	0	0	0
Dextrose	0	0	0	1	0
Pain medication	1	3	1	0	1
Polytrauma (*N* = 48)	(*N*= 5)	(*N*= 29)	(*N*= 10)	(*N*= 1)	(*N*= 3)
Mannitol	0	0	0	0	0
Pain medication	1	4	2	0	1
Splinting	0	0	0	0	0
Neck collar	0	0	0	0	0
Iv fluids	0	7	2	0	0
Airway management^a^	(*N*= 3)	(*N*= 6)	(*N*= 3)	(*N*= 2)	(*N*= 0)
Intubation	0	0	0	0	0
Suctioning	2	0	1	0	0
Oral airway	0	0	0	0	0
Total stabilized n/N (%)	7/26 (27)	34/257 (13.2)	6/60 (10)	1/16 (6.3)	2/9 (22)

^a^HC-health center, *DC* district hospital, *RC* regional hospital, *PRVT* Private hospitals, *OTHER* Police and Military hospitals

## Discussion

Stabilization of trauma patients prior to transfer improves outcome [15–17]. Our study provides a snapshot of initial stabilization of adult trauma patients provided by referring hospitals in Tanzania. We found that few patients had stabilization interventions before being referred to our ED. Moreover, most patients arrived more than 24-h after the injury. Lower facilities had a higher percentage of patients who received any form of initial stabilization compared to higher facilities, possibly as a result of patients passing from more than one facility prior to reaching MNH.

The majority of patients in our study were between 18 and 30 years old, with a predominance of males. These statistics are similar to other studies done previously in both HIC and LMIC illustrating the impact of trauma on a productive age group [7, 16–19]. Moreover the most common mechanism of injury was road traffic injuries, similar to these prior studies [6–8, 20, 21], However, motorcycle injuries were more common than MVA’s and falls in our study, which is similar to previous studies in LMIC, but less common in HICs [19, 22]; again these motorcycle accidents primarily affected an economically productive age group. With increasing urbanization in LIMC’s, motorcycles have become popular due to their lower cost and ability to maneuver through traffic. Poor roads and lack of adherence to traffic laws increases accidents [9, 23].

The primary purpose of this study was to investigate how well hospitals in Tanzania comply with initial stabilization of adult trauma patients according to the WHO essential trauma care guideline [24]. The majority of both head and extremity injury patients did not receive appropriate initial stabilization prior to referral to EMD MNH per WHO recommendations. We believe that this result is likely due to a knowledge gap among providers regarding initial stabilization and emergency care. Emergency medicine is a new field in Tanzania; there is only one full capacity emergency medicine department in the country, which opened in 2010. Lack of resources and equipment may also contribute to the low performance of stabilization measures [25]. Although not all patients were likely to require many of the specified interventions, there were clearly some areas where intervention was required but not done, patients with extremity injury needed splinting however it was not done properly or not done at all; patients with traumatic head injury should have cervical spine stabilization until they have been cleared; however none of them arrived to our hospital with a cervical spine protection and it was not recorded in the referral note. It is possible that additional procedures were done but not recorded. Even then, however, this can jeopardize patient care due to poor communication with the receiving facility.

Appropriate and timely initial stabilization in adult trauma patients is found to have reduced avoidable morbidity and mortality in developed countries with several studies providing evidence for recommendations regarding what should be performed, and how it should be done, in the initial stabilization of these patients [16, 18]. In order to translate this to LIMC’s, WHO created guidelines and a checklist. It is unclear if these facilities are aware of these guidelines and thus may benefit from the use of a checklist for referrals.

The delay in arrival to our ED after trauma is a result of the referral system currently established in the country and absence of formal pre hospital services [26]. It is important to note that the performance of stabilization measures was low across all levels of hospitals, including regional hospitals that are considered well resourced. The development of a prehospital system and designated trauma centers would avoid patients going to hospitals that do not contribute to care, and improve the timeliness of arrival at the appropriate facility. At the same time such a structure would reduce the burden of trauma on smaller hospitals which have more limited resources.

### Limitations

This was a single center study with a small sample size. However, MNH has the only full capacity emergency department in the country, and the patients studied were referred from multiple sites. Determination of whether stabilization was performed depended on documentation in the referral note and examination of the patient on arrival; thus some procedures may have been done but not recorded. In particular, this might be true of pain medication, as patients would not usually know if they received any.

## Conclusion

In this urban public emergency department in Tanzania, majority of trauma patients were referred from lower health facilities after 24-h of injury. Most did not receive initial trauma stabilization as recommended by the World Health Organisation guidelines. Future studies should identify barriers to pre-referral stabilization of adult trauma patients.

## Abbreviations

CRF: Case report form
ED: Emergency department
HIC: High-income countries
MNH: Muhimbili National Hospital
MOI: Muhimbili orthopaedic institute
MUHAS: Muhimbili University of Health and Allied Sciences
RTI: Road traffic Incident
WHO: World health organization
